# On a Case of Fracture of the Superior Maxilla and Its Treatment

**Published:** 1860-10

**Authors:** James Salter

**Affiliations:** Surgeon Dentist to Guy's Hospital.


### ARTICLE XX.

*On a Case of Fracture of the Superior Maxilla and its Treatment.* By JAMES SALTER, M. B., F. L. S., Surgeon Dentist to Guy's Hospital.

THE fracture of the jaw had occurred immediately behind the canine tooth, apparently passing vertically up to about the summit of the fangs of the pre-molars, and then extending at right angles horizontally backwards nearly, if not quite, to the posterior extremity of the bone. The anterior portion of the fractured bone was driven inwards upon the roof of the mouth to the extent of about the third of an inch; while the second molar tooth, implanted in the posterior portion, remained unmoved, the broken bone being, as it were, a radius, of which the second molar tooth was the fixed axis. The projection of bone into the roof of the mouth led to a corresponding depression on the outer side of the jaw, and this was somewhat apparent also on the face. Very little swelling had occurred, nor was much pain felt, except on manipulation. There was, however, considerable inconvenience experienced from the fact that the displaced teeth no longer opposed their fellows in the lower jaw by a normal "bite;" the cusps of the teeth, instead of interlocking with their inferior opponents, met them more or less on the points, and the mouth could not be satisfactorily closed.

On endeavoring to force the displaced bone into its proper situation, considerable pain was produced; it could not be got thoroughly into position, and when the pressure was removed the bone almost immediately resumed its former direction—projecting inwards. During this manipulation distinct crepitus could be felt.

To meet what appeared to me the requirements of this case, I adopted the following plan of treatment:—I prepared a gold plate to fit the interior of the mouth, so as to hold the displaced portion of bone as much as possible in position, thus far acting in every sense as a splint, but with the additional advantage that it was capable of repeated alterations from time to time so as still further to force outwards the inward projecting bone and teeth, as the patient could bear the pressure. I first took an accurate model of the mouth, and upon this the plate or splint, whichever it may be called, was constructed, embracing two of the firm teeth on the left side—namely, the first molar and second bicuspid; then, passing over the palate behind the incisors and canines, it was made to bear on the right side against the displaced teeth and bone, extending still further backwards and surrounding by a wire the second molar tooth, which had not been displaced. The plate was so constructed that it exercised pressure only on the bone and teeth which were displaced, and in an amount in proportion to the displacement—most upon the front of the broken bone and the first bicuspid, less upon the second bicuspid, and still less upon the first molar.

The accident occurred on the 17th of April; I first saw my patient on the 19th, and on the 23d the apparatus was applied. When the plate was first put on, it was very tight, and produced considerable pressure on the inner side of the fractured bone, causing, however, a sense of tension rather than pain. In a few hours this feeling passed off, and when, four days after, I next saw my patient, I found the position of the parts very much improved. This improvement was followed up, and still more advanced by altering the plate—making it wider, and by adding portions of gold within the crescentic excavations that received the teeth. From this date, the 27th April, my patient visited me about once a week, the plate having in that time ceased to produce any pressure, or exercise any influence requiring further alterations, till the 2d June, when, as I find from my notes, the maxillary arch was completely restored to its proper shape.

After this the plate was worn for many months—longer,

I think, than was required, the patient getting used to it, liking it, and feeling it a comfort and support. I cannot, therefore, name the precise date when osseous union may be said to have taken place, but it has taken place, retaining the fractured bone in a proper position. I do not see what other method of treatment could have succeeded in this case. Probably, had no mechanical support been employed, the already existing obliquity of the teeth would have been increased by the pressure of mastication, and certainly could not have been diminished; but by the means adopted, stability of the detached bone was secured, and its position gradually and progressively improved, till it assumed almost, if not quite, its original place.

As it seems to me, important as this mode of treating a fracture of the *upper* jaw may occasionally become, the same principle, as applied to the *lower* jaw, is of still greater service. Any displacement that accompanies fracture of the superior maxilla is simply a passive condition: there are no muscles attached to the upper jaw which can derange or draw out of place the broken bone. With the lower jaw, however, the case is very different. The inferior maxilla is a floating bone, to which many powerful muscles are attached, and those connected with the rotating movement of the jaw—namely, the pterygoids—having a strong lateral antagonistic action. Destroy the integrity of the maxillary arch by fracture, and that antagonism ceases: the muscles on either side act independently, and draw the points to which they are attached more or less to the mesial line; and this it is which causes the overlapping of the fractured ends of the bone, often so obstinate, and necessarily with it the traction of the chin towards the side on which the fracture has occurred in the usual cases, where the lesion is in the horizontal ramus on one side. A plate or splint, and for the lower jaw I prefer an ivory one, carved to fit the interior of the arch of the particular jaw, with small crescentic excavations for the existing teeth, when placed in the mouth, restores the integrity of the arch for the time being. It may be worn without inconvenience long enough to allow osseous union of the fractured ends, preventing displacement the while.

Not long since I was consulted by a professional friend in such a case as this. The fracture had occurred in the horizontal ramus, about the situation of the first molar tooth, and this tooth, as well as the second pre-molar, had long since been extracted, and the usual method of treatment—that of tying together teeth in the neighborhood of the fracture—could not be resorted to. The displacement of the jaw, and the consequent distortion of the face, were extreme. The fracture had occurred on the right side; the left pterygoid muscles, being no longer resisted by the right, drew over the mass of the bone—the left horizontal ramus, chin, and anterior portion of the right horizontal ramus—towards the right side, and to such an extent that the left lower canine tooth corresponded in position to the interval between the superior central incisors. My friend and myself agreed that the best and most promising plan would be to re-connect the broken and now separate portions of bone by an ivory arch or splint, within the jaw, adapted to its precise form, and attached to the remaining teeth. This plan was adopted with the happiest results, the ivory splint being worn till union had taken place, with scarcely any perceptible malposition of the jaw.

*British Journal of Dental Science.*

[The American Journal of '52 describes an apparatus used by Dr. Krider for the purpose of setting fractures of superior maxilla. From its being capable of application in all cases by means of adjusting screws and its immovable character when so adjusted, it has proved more practical than any other ever offered for this purpose. It was invented by C. Reinhardt of this city, surgical instrument maker.—*Ed.*]

